# Intake of medium-chain fatty acids induces myocardial oxidative stress and atrophy

**DOI:** 10.1186/s12944-018-0908-0

**Published:** 2018-11-17

**Authors:** Yoshihiro Miyagawa, Takuya Mori, Kei Goto, Isao Kawahara, Rina Fujiwara-Tani, Shingo Kishi, Takamitsu Sasaki, Kiyomu Fujii, Hitoshi Ohmori, Hiroki Kuniyasu

**Affiliations:** 1 Department of Molecular Pathology, Nara Medical University, 840 Shijo-cho, Kashihara, Nara 634-8521 Japan; 2 Division of Rehabilitation, Hanna Central Hospital, 741 Tawaraguchi-cho, Ikoma, 630-0243 Japan

**Keywords:** Medium-chain fatty acids, Oxidative stress, Cardiac dysfunction, Cachexia

## Abstract

**Background:**

Oral intake of medium-chain fatty acids (MCFAs) reportedly suppresses the accumulation of visceral fat and has antitumor effects in tumor-bearing animals. MCFAs penetrate the mitochondrial membrane in a carnitine shuttle-independent manner and are metabolized more quickly than long-chain fatty acids. Based on these characteristics, MCFAs may have pronounced effects in mitochondria-rich tissues, such as the myocardium. We examined the effect of oral intake of MCFAs on the heart.

**Methods:**

We fed BALB/c mice with a control diet supplemented with 0%, 2%, 5%, or 10% lauric acid (LAA; a 12-carbon saturated MCFA). After euthanasia, the hearts, both sides of quadriceps femoris muscle (QFM) and epididymal fat pad (EFP) were excised and weighed. Then myocardial tissue morphology, oxidative stress accumulation, and mitochondrial volume were observed by histological analysis. The expression levels of myosin light chain 1 were measured by ELISA.

**Results:**

There were no differences among the groups in food and calorie intake, but the intake of LAA increased with the dietary proportion. The 10%-LAA-fed mice experienced significant weight loss and became moribund on day 6. The body, cardiac and EFP weights of the mice fed 5% and 10% LAA were lower than those of the control group. And 10% LAA fed group showed significant decrease of the QFM weights. Protein analysis of the excised hearts revealed higher expression of myosin light chain 1 in the 5% group than in the control group. Histological examination of the hearts revealed myocardial atrophy and accumulation of oxidative stress in the 10% group. Fewer mitochondria were observed with increased LAA intake.

**Conclusions:**

Excessive LAA consumption may damage the myocardium and the damage might result from oxidative stress accumulation and cellular atrophy.

## Background

In recent years, the diet is worth noting because it is said that can provides medical or health benefits, including the prevention and/or treatment of a disease [1, 2]. Medium-chain fatty acids (MCFAs) are saturated fatty acids with 7 to 12 carbon atoms. Saturated fatty acids with 1 to 6 carbon atoms are defined as short-chain fatty acids and those with 13 or more are long-chain fatty acids [3]. In recent years, various health effects caused by MCFA intake have been reported, including improvement in metabolic syndrome [4], suppression of body fat accumulation [5], and induction of cancer cell apoptosis [6]. Unlike MCFAs, long-chain fatty acids require a transporter, such as CD36 or fatty acid-binding protein, to pass through the plasma membrane [7] and a carnitine shuttle to pass through the mitochondrial membrane [8]. MCFAs do not rely on membrane transporters for uptake into cells and can be directly transported to the mitochondrial intermembrane space without the carnitine shuttle [3, 9]. Therefore, cells readily utilize MCFAs for β-oxidation in mitochondria at rates corresponding to their blood concentrations [10, 11].

Myocardial cells contain abundant mitochondria, which occupy one-third of their cytoplasm. Under non-ischemic conditions, almost all ATP (>95%) is generated by oxidative phosphorylation (OXPHOS) in myocardial mitochondria [12]. In addition to glucose, fatty acids are used as substrates (60%–80%) for OXPHOS in cardiomyocytes [13]. Based on these characteristics, MCFAs are thought to play important roles in cardiomyocytes and function as muscle cell energy sources.

In the present study, we examined the effects of MCFAs on the heart using a mouse model.

## Materials and Methods

### Animals

We purchased 12 male BALB/c mice (5 weeks old; mean body weight, 21.7 ± 0.8 g) from SLC Japan (Shizuoka, Japan). The animals were maintained in a pathogen-free animal facility on a 12-h/12-h light/dark cycle in a temperature (22°C)- and humidity-controlled environment. Animal maintenance and experiments were conducted in accordance with the institutional guidelines approved by the Committee for Animal Experimentation of Nara Medical University (Kashihara, Japan), consistent with the current regulations and standards of the Japanese Ministry of Health, Labor and Welfare (approval number 12023). Animals acclimated to their housing for 7 days before the start of the experiment.

### Animal model

Lauric acid (LAA, a 12-carbon saturated MCFA; Tokyo Chemical Industry, Tokyo, Japan) was used as the model MCFA. Mouse chow was prepared by mixing 0, 2, 5, and 10% LAA with the control CE-2 diet (CLEA Japan, Tokyo, Japan), which contained 5% crude fat derived mainly from soy bean oil. The mice were divided into 4 groups and fed 1 of the 4 diets. We measured body weight and food intake and exchanged food each day until the end of the experiment. The 10% group had marked weight loss and were moribund on day 6 and the 5% group showed decreased activity on day 13, so the mice were euthanized at those time points. The 0% (control) and 2% groups were maintained for 15 days, then euthanized. After euthanasia, the hearts, both sides of quadriceps femoris muscle (QFM) and epididymal fat pad (EFP) were excised and weighed. The hearts were divided into 2 parts at two-fifths the distance from the apex of the heart; the upper part was used for histological analysis and the lower part was used to analyze protein expression. We calculated daily food, LAA, and calorie intake per mouse from the total intake of 3 mice in each group.

### Histological analysis

Myocardial tissues were fixed in 4% paraformaldehyde, dehydrated, then embedded in paraffin. We sliced 3-μm sections, then performed hematoxylin and eosin (H&E) and immunohistochemical staining to observe tissue morphology, oxidative stress accumulation, and mitochondrial volume in the myocardial tissues. We used an anti-8-hydroxy-2′-deoxyguanosine (8-OHdG) antibody (Japan Institute for the Control of Aging, NIKKEN SEIL, Shizuoka, Japan) to assess the accumulation of oxidative stress in the nuclei and an anti-4-hydroxy-2-nonenal (4-HNE) antibody (Japan Institute for the Control of Aging, NIKKEN SEIL) to assess it in the cytoplasm. We used an anti-leucine zipper-EF-hand containing transmembrane protein 1 (Letm1) antibody (16024-1-AP; Proteintech Group, Rosemont, IL, United States) to observe mitochondria in myocardial tissue. The primary antibodies were used at 1.0 μg/mL and secondary antibodies (Medical and Biological Laboratories, Nagoya, Japan) were used at 0.2 μg/mL. Tissue sections were color-developed with diamine benzidine hydrochloride (DAKO, Glastrup, Denmark) and counterstained with Meyer’s hematoxylin (Sigma-Aldrich Chemical, St. Louis, MO, United States) to visualize nuclei. The numbers of cells positive for oxidative stress markers were counted and compared between groups. The histological analyses were verified using a fluorescence microscope (BZ-X710, Keyence, Osaka, Japan).

### Analysis of protein expression

The lower parts of the excised hearts were pelleted at low temperature (<4°C), then the proteins were extracted with RIPA buffer (Thermo Fisher Scientific, Tokyo, Japan). We quantified total protein for each sample, then measured the levels of myosin light chain 1 (Myl1) and mitochondria. The levels of Myl1 were measured using the Mouse Myosin light chain 1/3, skeletal muscle isoform (MYL1) ELISA kit (CSB-EL015305MO; Cusabio Biotech, Houston, TX, United States), according to the manufacturer’s instructions. The data were normalized by cell number after quantifying the amount of protein.

The levels of mitochondria were measured by western blot analysis. Lysates were separated by 10.0% sodium dodecyl sulfate-polyacrylamide gel electrophoresis and transferred to nitrocellulose membranes. The membranes were incubated with a primary antibody specific to Letm1 (16024-1-AP; Proteintech Group), then a peroxidase-conjugated IgG antibody (P0217; Dako). We used an anti-β-actin antibody to assess the levels of protein loaded per lane (clone sc47778, catalog number C0817; Santa Cruz Biotechnology, Santa Cruz, CA, United States). Immune complexes were visualized using a Fusion Solo (M&S Instruments, Osaka, Japan).

### Statistical analysis

Statistical significance was calculated using two-tailed Fisher's exact test, χ^2^ test, and unpaired Student’s t-test using InStat software (version 3.0; GraphPad Software, La Jolla, CA, United States). Data are expressed as the mean ± standard deviation of 3 independent experiments. Differences for which *P*<0.05 (two-sided) were considered statistically significant.

## Results

### Effects of oral intake of LAA on body, quadriceps muscle and EFP weight

**Fig. 1:**
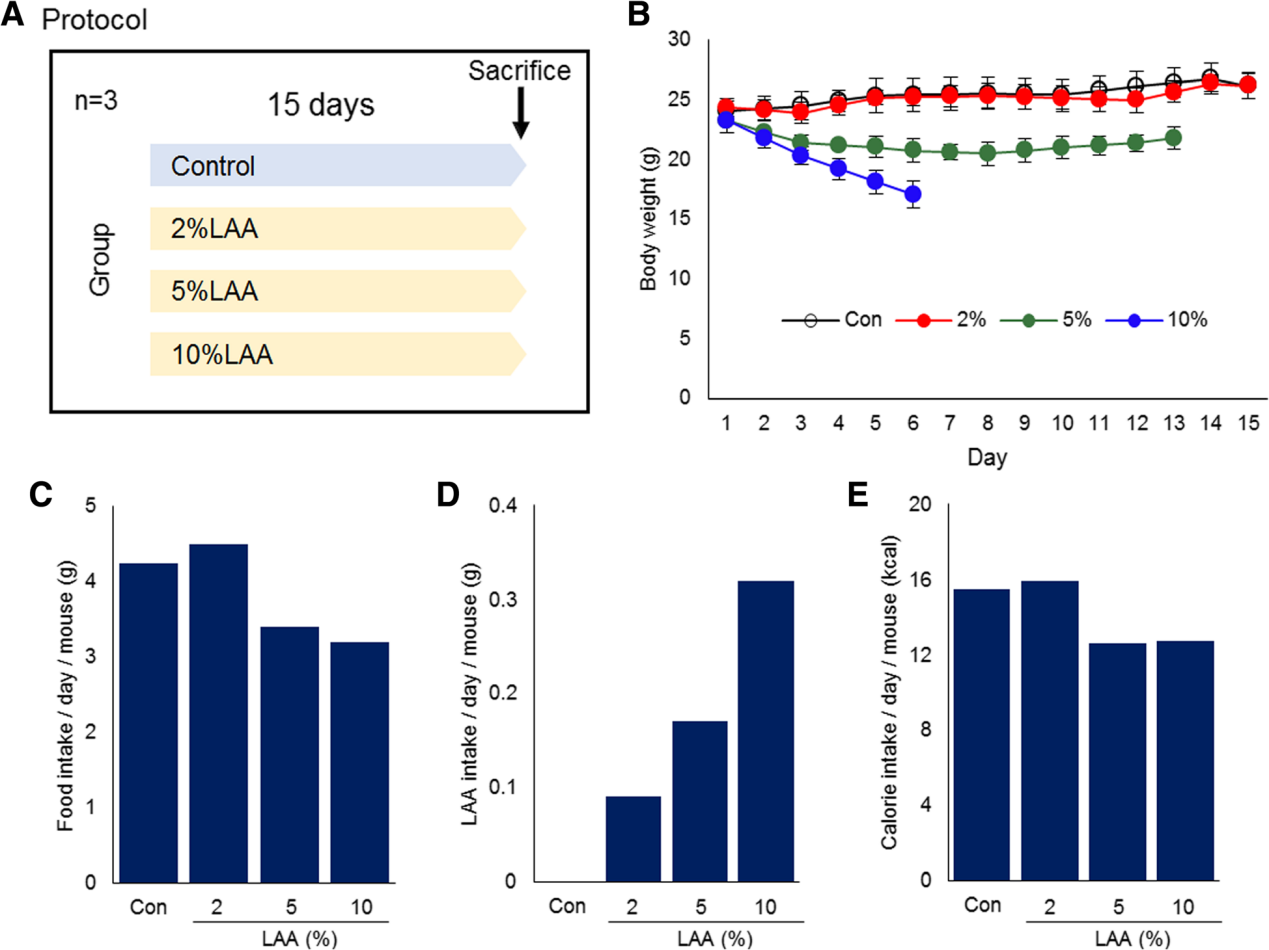
LAA intake mouse model. **a** Experimental protocol. **b** Body weight changes. The data are expressed as the mean ± standard deviation. **c-e** Food, LAA, and calorie intake. The daily intake per mouse was calculated from the total intake of 3 mice in each group. Con: control; LAA: lauric acid

First, we assessed the effect of LAA in the diet on body weight and total daily dietary intake (Fig. 1). We did not observe differences in body weight between the control and 2% group. However, the 5% and 10% groups lost body weight after starting the diet; their average body and EFP weights at the time of euthanasia were significantly lower than that of the control group (*p*<0.01) (Fig. 1b, Table 1). In addition, in the 10% group, the weight of QFM were significantly lower than that of the control (*p*<0.01) (Table 1). Total diet and calorie intake were somewhat lower in the 5% and 10% groups than in the control group (Fig. 1c, e). The intake of LAA increased based on the concentration in the diet (Fig. 1d).

**Table 1:** The body, QFM and EAA weight of mouse at euthanasia in each groups

	Control	2%LAA	5%LAA	10%LAA
Body weight (g)	26.2±1.1*	26.2±1.1	21.8±1.0**	17.1±1.1***
QFM weight (g)	0.20±0.03	0.22±0.01	0.18±0.01	0.14±0.02**
EFP weight (g)	0.25±0.04	0.22±0.01	0.10±0.03***	0.03±0.01***

*Data are expressed an mean ± standard deviation

**p < 0.01, vs the control group

***p < 0.001, vs the control group. *QFM* quadriceps femoris muscle; *EFP  *epididymal fat pad

### Heart morphology after LAA consumption

**Fig. 2:**
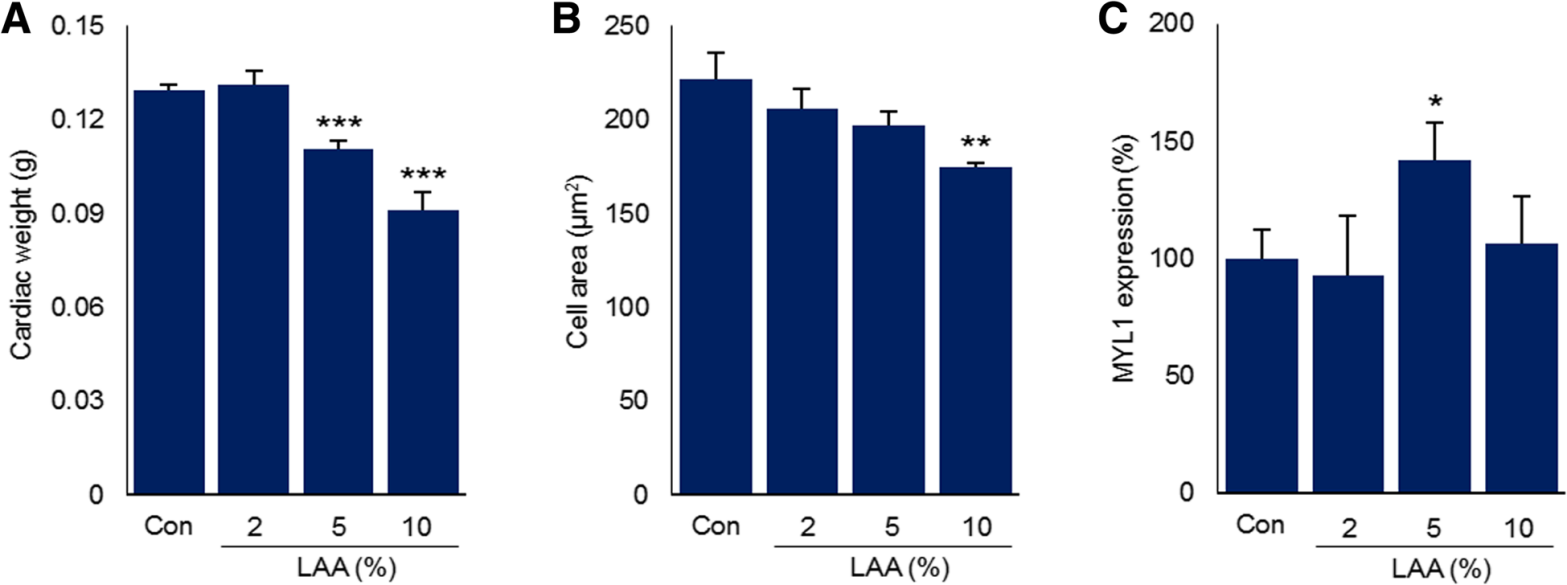
Influence of LAA intake on the heart. **a** Cardiac weight. **b** Cardiomyocyte cell area. Histopathological specimens were observed under a microscope. The number of cells per unit area was measured. **c** Expression of Myl1 in the excised myocardial tissue. Myl1 expression after LAA intake is presented relative to the expression in the control, which was set as 100%. The data are expressed as the mean ± standard deviation. *p < 0.05, vs the control group; **p < 0.01, vs the control group; ***p < 0.001, vs the control group. Con: control; LAA: lauric acid; Myl1: myosin light chain

**Fig. 3:**
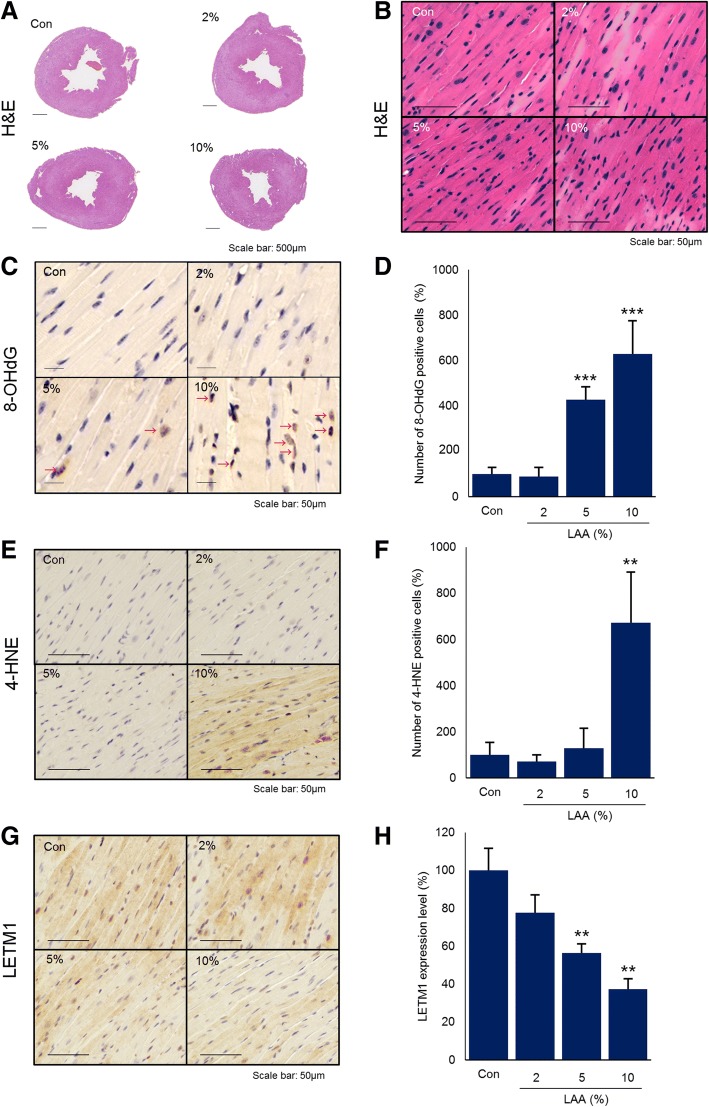
Heart morphology and expression of oxidative stress- and mitochondria-related proteins in cardiomyocytes. **a** and **b** Low- and high-power images of H&E staining in histological specimens, respectively. **c-f** Immunohistochemical staining for oxidative stress in nuclei (**c**) and cytoplasm (**e**) and graphs of the numbers of positive cells (**d** and **f**). Arrow indicates positive nucleus (**c**). **g** and **h** Immunohistochemical staining for mitochondria with a Letm1 antibody (**g**) and Letm1 expression quantified by western blotting (**h**). These data are expressed relative to the control, which was set as 100%. The data are expressed as the mean ± standard deviation. **p < 0.01, vs the control group; ***p < 0.001, vs the control group. H&E: hematoxylin and eosin; 8-OHdG: 8-hydroxy-2’-deoxyguanosine; 4-HNE: 4-hydroxy-2-nonenal; Con: control; Letm1: leucine zipper-EF-hand containing transmembrane protein 1

The hearts of the 5% and 10% groups weighed significantly less than those of the control group (*p*<0.001) (Fig. 2a). In addition, the hearts of the 10% group exhibited significant cell atrophy compared with those of the control group (*p*<0.01) (Fig. 2b, Fig. 3b). We observed a significant inverse correlation (*r*=-0.99, *p*<0.01) between LAA intake and cardiomyocyte area.

### Effects of LAA on myocardial structural protein expression

We measured the expression of Myl1 by enzyme-linked immunosorbent assay as an indicator of myocardial maturity. Myl1 expression was significantly higher in the 5% group than in the control group (*p*<0.05). On the other hand, the expression in the 2% and 10% groups did not significantly differ from that in the control group (Fig. 2c).

### Impact of LAA on oxidative stress in myocardial tissue

Immunohistochemical staining with an anti-8-OHdG antibody revealed no accumulation of oxidative stress in the myocardial tissues of the control and 2% groups. In contrast, we observed the accumulation of oxidative stress in the nuclei of the myocardial tissues of the 5 and 10% groups (Fig. 3c). In addition, immunohistochemical staining with an anti-4-HNE antibody revealed a similar accumulation of oxidative stress in the cytoplasm of the myocardial tissue in the 10% group (Fig. 3e). We counted the number of 8-OHdG- and 4-HNE-positive cells per visual field. We found significantly more 8-OHdG-positive cells in the 5% and 10% groups than in the control group (*p*<0.001) and significantly more 4-HNE-positive cells in the 10% group than in the control group (*p*<0.01) (Fig. 3d, f).

### Effects of LAA on the mitochondrial volume in myocardial tissue

Based on immunohistochemical staining for Letm1, we observed lower mitochondrial volume in the 5 and 10% groups than in the control group (Fig. 3g). In addition, western blot analysis revealed no significant difference between the control and 2% group, but a significant decrease in mitochondrial volume in the 5 and 10% group compared to in the control group (*p*<0.01) (Fig. 3h).

## Discussion

Our study had 3 major findings. First, myocardial cell atrophy correlated with the dose of LAA. Second, oxidative stress accumulated in cardiomyocytes upon excessive administration of LAA. Third, mitochondrial volume in the myocardium was decreased by administration of LAA.

In previous studies of MCFA administration, atrophy of the myocardium was not observed after ingestion of food in which MCFAs comprised 12% of caloric intake [4]. In addition, in a spontaneously hypertensive rat model, myocardial hypertrophy is suppressed by administration of a 5%-MCFA diet for 4 months [14]. On the other hand, high concentrations of MCFAs (40% of calorie intake) cause increased hepatic synthesis of fatty acids from MCFA through de novo synthesis and/or chain elongation and desaturation [15]; however, the reports do not mention the morphology of myocardial cells. LAA can be directly incorporated into mitochondria and undergo β-oxidation without a special transporter or carnitine shuttle [3, 9]. As a result, it may cause excessive OXPHOS in mitochondria and increase oxidative stress, thereby enhancing the myocardial catabolism pathway.

In this study, we investigated the accumulation of oxidative stress in myocardial tissue by measuring 4-HNE and 8-OHdG. 4-HNE is a secondary product of lipoperoxidation, can form protein adducts and modify cell signaling [16], is considered a biomarker of oxidative stress, and is one of the most reactive aldehydes [16, 17]. In nuclear and mitochondrial DNA, 8-OHdG is a predominant form of free radical-induced oxidative lesion and has, therefore, been widely used as a biomarker for oxidative stress and carcinogenesis [18]. We found that both 4-HNE and 8-OHdG accumulated in cardiomyocytes in the 5% and 10% groups. The accumulation of oxidative stress in muscle cells activates myostatin and atrogin-1 [19], thereby enhancing the ubiquitin–proteasome system and promoting the degradation of constitutive muscle proteins [20]. These findings suggest that a high concentration of LAA increases the accumulation of oxidative stress, increases the catabolic pathway in cardiomyocytes, and induces cellular atrophy.

The accumulation of 4-HNE decreases mitochondrial function by modifying and inactivating tricarboxylic acid cycle-related proteins in mitochondria and other organelles [21]. Furthermore, the accumulation of 8-OHdG causes a decrease in mitochondrial DNA copy number and inhibits mitochondrial biogenesis [22]. In this study, oxidative stress was induced by a high concentration of LAA, which likely caused the decrease in the mitochondrial volume of the myocardium. Although mitochondrial impairment has been reported to induce apoptosis through the release of cytochrome c [23], we did not observe an increase in apoptosis in this study. These results suggest that administration of excessive LAA caused mitochondrial damage that produced myocardial damage by inducing catabolism of intracellular proteins by oxidative stress rather than inducing cardiomyocyte apoptosis.

In literature, detrimental effect of fatty acids is thought to be a lipotoxicity, which is provided by lipid intermediates [24]. Toxic fatty acid metabolites such as ceramides, diacylglycerols, long-chain acyl-CoAs, and acylcarnitines accumulate in cardiomyocytes to cause their dysfunction. In cancer cells, LAA provided marked oxidative stress to induce apoptosis, whereas linoleic acid or elaidic acid did not [25–27]. The findings suggest that LAA might possess different effect on production of oxidative stress in comparison with long-chain fatty acids. It is needed to examine the toxic metabolites in the myocardium of LAA-fed mice.

This study revealed that administration of excessive LAA causes myocardial injury. However, 2% LAA can be administered without inducing obvious myocardial damage. Expression of Myl1, which reflects myocardial maturity, increased after feeding with 5% LAA, the same concentration at which myocardial atrophy was observed. Further studies using different maturation markers are needed, particularly to examine the effects of 2% LAA on myocardial maturity. Focusing on the weight loss due to ingestion of LAA, cardiac cachexia is defined as weight loss of skeletal muscle and adipose tissue [28], and the current model may be available as an cardiac cachexia model. It has also been reported that cancer cell proliferation is suppressed by LAA administration [25]. That finding and the results of this study suggest that an appropriate dose of LAA may be applicable for anticancer treatment. In this way, LAA have not only the role of supplementing nutrition but also the possibility that it can be useful for the improvement and prevention of diseases by changing the behavior of cells.

## Conclusion

We found that by excessive LAA consumption, oxidative stress accumulates in cardiomyocytes and the cells become atrophied causing myocardial damage. Oxidative stress accumulated in both nuclei and cytoplasm, and the mitochondrial volume was decreased. This LAA fed mouse model might be useful as a cardiac cachexia model. In future studies, we will examine the appropriate doses of LAA in greater detail and continue to investigate its effects in disease models with the aim of developing a strategy for clinical application.

## Abbreviations

4-HNE: 4-hydroxy-2-nonenal
8-OHdG: 8-hydroxy-2′-deoxyguanosine
EFP: Epididymal fat pad
H&E: Hematoxylin and eosin
LAA: Lauric acid
Letm1: Leucine zipper-EF-hand containing transmembrane protein 1
MCFAs: Medium-chain fatty acids
Myl1: Myosin light chain 1
OXPHOS: Oxidative phosphorylation
QFM: Quadriceps femoris muscle
